# Molecular Self-Assembly at Metal-Electrolyte Interfaces

**DOI:** 10.3390/ijms14034498

**Published:** 2013-02-25

**Authors:** Thanh Hai Phan, Klaus Wandelt

**Affiliations:** 1 Institute of Physical and Theoretical Chemistry, University of Bonn, Wegelerstr, 12, D-53115 Bonn, Germany; 2 Institute of Experimental Physics, Plaza Maxa Borna 9, 50-204 Wroclaw, Poland

**Keywords:** self-assembly, porphyrin, viologen, cyclic voltammogram, scanning tunneling microscopy, X-ray photoelectron spectroscopy

## Abstract

The self-assembly of molecular layers has become an important strategy in modern design of functional materials. However, in particular, large organic molecules may no longer be sufficiently volatile to be deposited by vapor deposition. In this case, deposition from solution may be a promising route; in ionic form, these molecules may even be soluble in water. In this contribution, we present and discuss results on the electrochemical deposition of viologen- and porphyrin molecules as well as their co-adsorption on chloride modified Cu(100) and Cu(111) single crystal electrode surfaces from aqueous acidic solutions. Using *in situ* techniques like cyclic voltametry and high resolution scanning tunneling microscopy, as well as ex-situ photoelectron spectroscopy data the highly ordered self-assembled organic layers are characterized with respect to their electrochemical behavior, lateral order and inner conformation as well as phase transitions thereof as a function of their redox-state and the symmetry of the substrate. As a result, detailed structure models are derived and are discussed in terms of the prevailing interactions.

## 1. Introduction

The self-assembly of molecular layers has become an important strategy in modern design of functional materials. However, in particular, large, thermally unstable organic molecules may not be intact volatile to be deposited by vapor deposition. In this case, deposition from solution may be a promising route, and in ionic form, these molecules may even be soluble in water. In ionic form, the molecules become subject to the laws of electrostatics and electrochemistry. Among all possible molecule-molecule and molecule-substrate interactions, electrostatic forces will therefore play an important role. It may thus be considered an advantage of electrochemical deposition that these electrostatic interactions can be “tuned” by the electrochemical potential, *i.e.*, the charge state, of the electrode surface in two ways. Firstly, the mere charge density at the electrode surface will influence the attractive forces acting between the adsorbed ions and the substrate. Secondly, depending on the electrode potential the adsorbed molecular ions may undergo redox-reactions due to charge transfer processes between them and the surface. The concomitant change in their own charge state will influence the intermolecular interactions within the adsorbed layer. Both the potential dependent electrostatic adsorbate-substrate interactions as well as the change in electrostatic forces between the adsorbed molecular ions due to redox-transitions are expected to influence the structure of the electrochemically deposited molecular layers.

Among others, porphyrin and viologen molecules have emerged as model systems for the design and fabrication of molecular architectures of nanometer-size and the characterization of their formation mechanisms [1–4], because of their interesting properties as well as potential and multidisciplinary applications in physics, chemistry and biology, *i.e.*, molecular devices, electrocatalysis, and photosynthesis [5,6].

Porphyrins and related derivatives are known to be relevant in metalloproteins and chlorophyll as active centers [7]. Detailed research on porphyrin molecules may therefore lead to a better understanding of their role in electrochemical and photochemical processes such as dioxygen storage and photosynthesis. Especially, porphyrin thin films have been intensively investigated with respect to their activity as electrocatalysts in electrochemical reactions such as the reduction of O_2_ for the development of efficient fuel cells [8–10]. In particular, ordered thin films of porphyrin derivatives forming at solid/electrolyte interfaces have received particular attention [11]. Miyake *et al.*[11] have studied molecular arrays of a series of meso-tetra-substituted porphyrins on an HOPG surface in electrochemical environment by using electrochemical scanning tunneling microscopy (EC-STM). As a result, 5,10,15-*tris*-(4-octadecyloxyphenyl)-20-(4-pyridyl)porphyrin molecules were found to form slightly undulating rows, while more kinky rows were observed with 5-(4-carboxyphenyl)-10,15,20-*tris*(4-octadecyloxyphenyl)porphyrin. Similarly, the ordered adlayers of three water soluble porphyrin molecules, *i.e.*, iron(III) protoporphyrin, zinc(II) protoporphyrin, and metal free protoporphyrin attached to the basal plane of graphite were successfully studied in aqueous solution by Tao *et al.*[12,13] using both STM and AFM measurements. Since simple anions like halides do not adsorb on HOPG, the molecular structures in this case are only influenced by the charge state of the bare HOPG surface.

With respect to metal surfaces He *et al.*[14,15] have investigated the lateral ordering and surface mobility of 5,10,15,20-tetrakis (*N*-methyl-4-pyridinium)-21H,23H-porphine (H_2_TMPyP) molecules (see Figure 7) adsorbed at the electrochemical interface of a bare Au(111) surface as a function of potential. Itaya and his coworkers reported on the self-assembly of the water soluble [H_2_TMPyP]^4+^ cations on anion modified metal surfaces. Highly ordered adlayers of [H_2_TMPyP]^4+^ cations were formed on iodide terminated Au(111), Ag(111), Pt(111), and Pt(100) [16–18] surfaces as well as on the sulfur modified Au(111) [19] electrode. More recently, Hai *et al*. found that highly ordered adlayers of the [H_2_TMPyP]^4+^ molecular ions were also formed on sulfate [20], and iodide [21] modified copper electrodes.

Likewise, viologen based thin films have also attracted much attentions due to their widespread applications in electronic devices [22–25] as well as light harvesting operators [26]. In electrochemical environments viologen species are well known to exist in three oxidation states, namely as dicationic species (V^2+^), radical monocations V^+•^ and uncharged viologen molecules V^0^, respectively [27,28]. Charge transfer reactions within adsorbed ordered layers of viologens as a function of the electrode potential are reported to result in reversible structure transitions [29,30]. For instance, adsorption of 2.2′-bipyridine molecules on a Au(111) surfaces in HClO_4_ acidic solution was reported by Wandlowski *et al.*[29]. Later, Diao *et al*. [30] found that 4,4′-Bipyridine molecules strongly adsorb on the bare Cu(111) electrode and form, potential dependent, two distinct ordered phases depending on their oxidation state. As mentioned above, a detailed characterization of such charge transfer governed phase transitions under electrochemical conditions yields insight into the molecule-molecule and molecule-substrate interactions and, thereby, the possibility to govern the structure formation *via* the tunable surface charge density.

Moreover, competitive co-adsorption of two or more different organic molecules has also been investigated [31–35], because the resultant new intermolecular interactions between unlike molecules opens new degrees of freedom for the design of supramolecular nano-architectures. For example, two-component films consisting of porphyrin and phthalocyanine derivatives have been examined by Kobayashi [31], Hipps [32,33] and recently Itaya [34,35], but systematic studies in this direction are still scarce.

Characterization of the electrochemical and structural properties as well as phase transitions of such ordered adlayers on metal electrodes as a function of their redox-state, the pre-adsorbed anion species and the symmetry of the substrate is therefore of importance in understanding and controlling the properties of both single molecular and bimolecular adlayers in aqueous solution under electrochemical conditions.

In the present work a survey is given about the electrochemical properties, surface structures and phase transitions of a pure viologen and a pure porphyrin layer as well as of mixed layers of both molecular species forming on chloride modified Cu(100) and Cu(111) electrode surfaces in aqueous solution under electrochemical conditions. We first concentrate on the electrochemical properties as well as the molecular self-assembly of one representative species of each of the two classes of molecules, namely 1,1′-dibenzyl-4,4′-bipyridinium, *i.e*., -viologen, and 5,10,15,20-Tetrakis (4-trimethyl ammonium phenyl) porphyrin tetra (p-toluenesulfonate) molecules, abbreviated as DBV and H_2_TTMAPP, respectively. Subsequently we also investigate the properties of binary adlayers consisting of both coadsorbed species. Besides the standard electrochemical method of cyclic voltametry [36,37] also state-of-the-art surface analytical methods are employed, namely *in situ* Electrochemical Scanning Tunneling Microscopy (EC-STM) as a local structure sensitive technique and *ex situ* X-ray Photoelectron Spectroscopy (XPS) as a chemical probe. In particular, EC-STM enables a direct visualization of structural changes within the adsorbed molecular adlayers caused by either mere changes of the electrode potential or by surface reactions due to electron transfer processes.

## 2. Results and Discussion

### 2.1. Structures of the Anion Modified Cu(100) and Cu(111) Substrates

**Figure 1:**
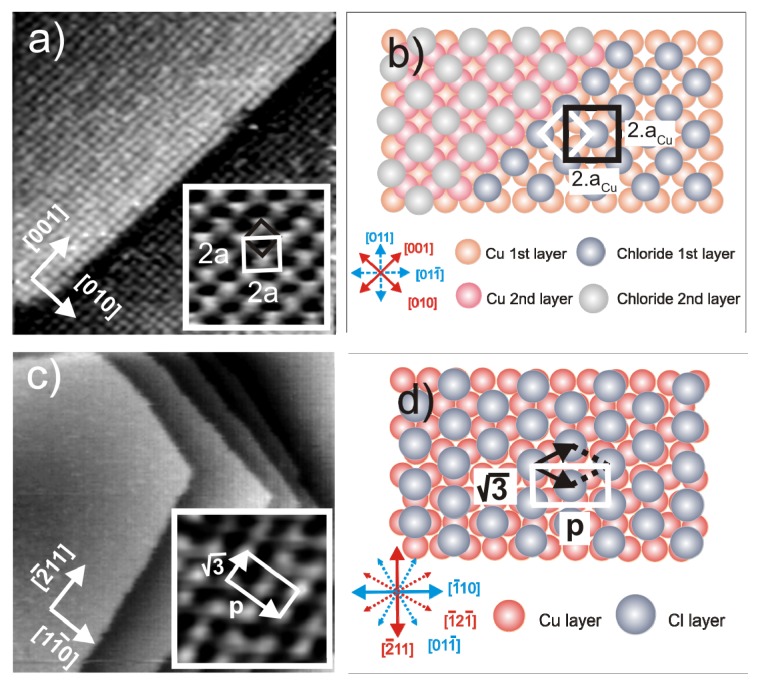
(**a**) Typical in situ STM images of a chloride covered Cu(100) electrode surface; copper steps are preferentially aligned parallel to the close packed Cl rows. Large scale image: 9.92 nm × 9.92 nm, *U*_b_ = +40 mV, *I*_t_ = 5 nA, *E* = +20 mV; inset: 4.37 nm × 4.37 nm, *U*_b_ = +20 mV, *I*_t_ = 2.5 nA, *E* = −10 mV; (**b**) Structural model of the *c*(2 × 2) – *Cl* adlayer on Cu(100). (**c**) Chloride modified Cu(111) surface. Large scale image: 32.4 nm × 32.4 nm, *U*_b_ = +220 mV, *I*_t_ = 0.1 nA, *E* = −10 mV, and inset image 2.43 nm × 2.43 nm, *I*_t_ = 2.0 nA, *U*_b_ = 75 mV, *E* = 0.0 mV; (**d**) Hard-sphere model of the *c*(*p* × √3) – *Cl* adlayer on Cu(111).

**Figure 2:**
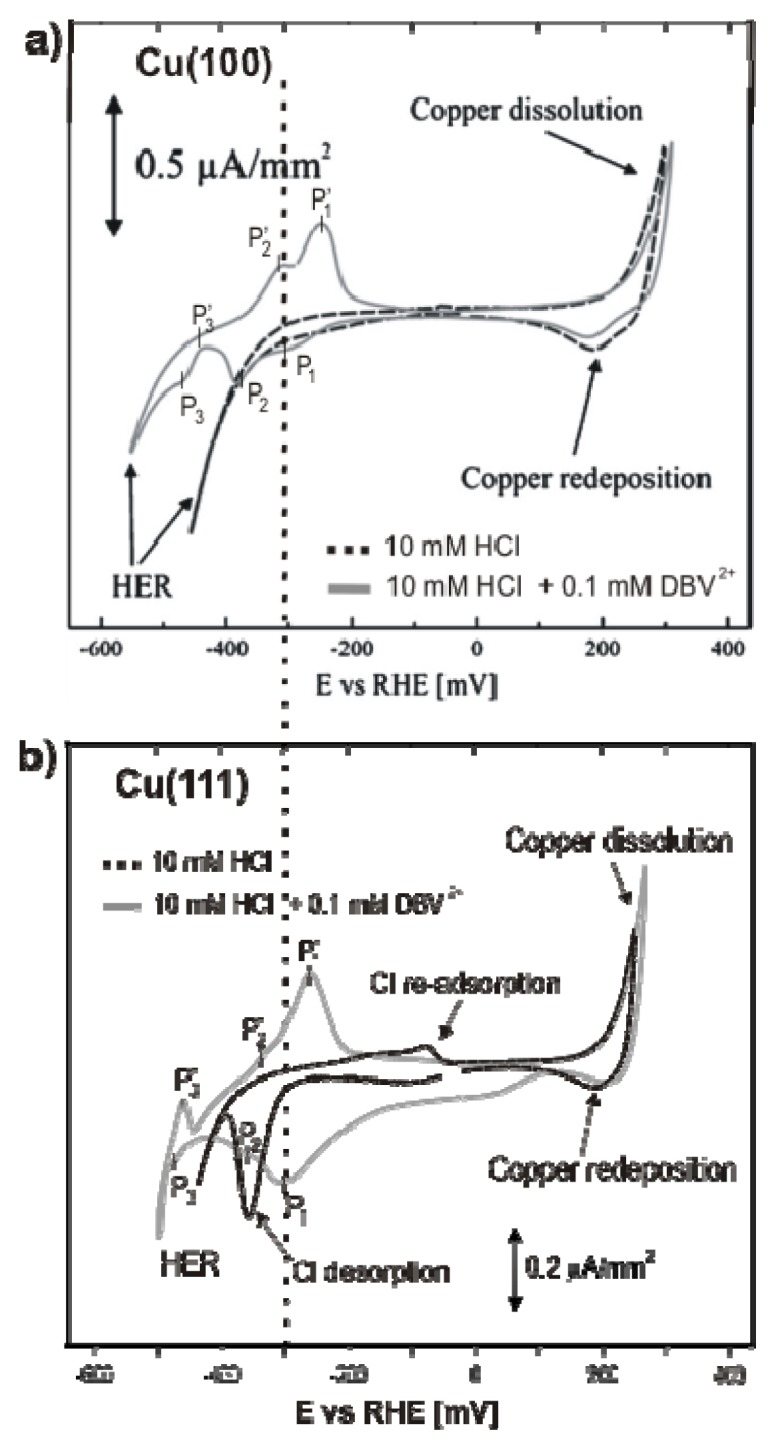
Representative steady-state cyclic voltammograms (CVs) of (**a**) Cu(100) and (**b**) Cu(111) in pure 10 mM HCl solution (dashed black curves) and in (10 mM HCl + 0.1 mM DBV) electrolyte (grey curves), respectively; d*E*/d*t* = 10 mV/s. Figure 2a is reproduced with permission from [38].

Chloride anions are known to adsorb strongly on copper electrodes forming well ordered phases. In case of a Cu(100) electrode surface, a typical *c*(2 × 2) – *Cl* phase [38–42] is readily formed in the whole potential regime between the copper dissolution reaction (CDR) and the on-set of the hydrogen evolution reaction (HER). A steady state cyclic volammogram of Cu(100) in pure 10 mM HCl solution is included in Figure 2a. High resolution STM images of the fourfold symmetric chloride structure adsorbed at positive potentials E on top of a Cu(100) electrode surface and a hard-sphere model of the resulting *c*(2 × 2) – *Cl* layer are presented in Figure 1a,b. Exploiting the bias (*U*_b_) dependence of the STM images it is possible to distinguish quasi-spectroscopically between contributions originating from chloride and copper to the registered tunneling current, respectively, and, thereby, to prove that the chloride anions occupy hollow sites [42].

In contrast, on the Cu(111) electrode, the adsorbed chloride phase is a function of the electrode potential. A steady state cyclic voltammogram of Cu(111) in 10 mM HCl solution is included in Figure 2b. Here chloride desorbs at ~(−350 mV) and re-adsorbs at ~(−80 mV) *vs.* RHE. Whereas a simple (√3 × √3) R30° chloride structure is observed in the regime of negative potentials near peak P_2_[43–45], a uniaxial incommensurate *c*(*p* × √3), “quasi-hexagonal” phase with an additional long-range height modulation is obtained in the positive potential regime after the re-adsorption peak [46,47]. In this work, the *c*(*p* × √3) – *Cl* covered Cu(111) surface will be used as a substrate for the adsorption of the positively charged organic molecules. Figures 1c,d show representative atomic level STM images of the quasi-threefold symmetric chloride lattice on top of the Cu(111) electrode surface as well as a model of this adsorbed phase.

On both surfaces, the adsorbed chloride anions induce a preferential orientation of the substrate steps parallel to the close-packed chloride rows (Figure 1a,c and [4]). Consequently, the directions of the chloride rows are rotated by an angle of 45° [48] and 30° [20,45] with respect to the main symmetry axes of the underlying Cu(100) and Cu(111) surfaces, respectively. As a result, there are two *c*(2 × 2) – *Cl* rotational domains on Cu(100) and three *c*(*p* × √3) – *Cl* domains on Cu(111), which are rotated by 90° (120°) with respect to each other, reflecting the electrode symmetry.

Besides these mere structure and symmetry aspects, recent *in situ* XRD measurements in combination with theoretical calculations have provided additional information about the charge state of the chloride precovered copper surfaces [49,50]. The XRD measurements yielded a Cu-Cl interlayer spacing of 1.88 Å (1.86 Å) for the Cu(100) (Cu(111)) surface, which corresponds to a Cu–Cl bond length of 2.61 Å (2.40 Å) on Cu(100) (Cu(111)), respectively. In both cases the bond length between the Cu surface atoms and the adsorbed Cl anions is longer than the Cu–Cl bond length found in solid copper chloride compounds as well as for chloride adsorbed on the two surfaces in UHV [51]. This is strong indication for a more ionic bond between Cu and the adsorbed Cl anions in solution, in agreement with theoretical calculations, and makes the chloride modified Cu surfaces good candidates for the electrochemical deposition of the positively charged viologen and porphyrin cations on the negatively charged Cl/Cu electrode surfaces. By contrast, pre-adsorbed iodide layers are found to be bound more covalently, making their electrostatic interaction with co-adsorbed porphyrin cations less strong [52].

### 2.2. DBV Adlayers on Chloride Modified Cu(100) and Cu(111)

#### 2.2.1. Electrochemistry

Figure 2 presents the typical steady-state cyclic voltammograms of Cu(100) and Cu(111) in pure electrolyte (10 mM HCl) and in an electrolyte containing the redox active viologen (working electrolyte: 10 mM HCl + 1 mM DBVCl_2_). As mentioned in the previous section, the potential window of the copper electrodes in the pure electrolyte is limited by the copper dissolution reaction (CDR) at the anodic limit and the hydrogen evolution reaction (HER) at the cathodic limit (dash black curves). A drastic change in the voltammetric behavior is observed in the DBV-containing solution (grey curve), namely three new pronounced pairs of peaks appear and the HER is shifted toward lower potentials. The shift of the HER is probably caused by viologen molecules blocking the most reactive surface sites for this reaction. The additional current waves are assigned to viologen related redox processes, as well as an order/disorder phase transition due to chloride adsorption/desorption [53]. It is well known that the viologen dications (V^2+^) undergo two separate one-electron transfer steps in electrochemical environment forming first the respective viologen radical mono-cations (V^+•^) and then the uncharged species (V^0^). While the uncharged viologen species accumulate at the electrode surface due to their hydrophobic properties [4] the dication and radical cation species are soluble in aqueous solutions.

Specifically, the three quasi-reversible pairs of peaks, P_1_/P_1_′, P_2_/P_2_′, P_3_/P_3_′, respectively, observed in Figure 2a,b with decreasing potential, are assigned to the reduction of dicationic DBV^2+^ to the corresponding radical mono-cations DBV^•+^, an order/disorder phase transition due to chloride desorption/adsorption, and finally the precipitation/dissolution of an adsorbed film of neutral viologen molecules, respectively [38,54,55].

The complex appearance of the peaks, in particular their relative intensities, derives from the superposition between “solution” processes and “surface limited” reactions as follows:

The reduction/oxidation of preadsorbed viologen and/or viologen species from solution is described by:

(1) $$DBV^{2+}+e^{-}\Leftrightarrow DBV^{•+}$$

This reduced species may form dimers- and/or polymers depending on whether the process occurs in solution or on the surface:

(2) $$2(or\;n)DBV^{•+}\Leftrightarrow {\left[DBV_{2}\right]}^{2+}\left(or{\left[DBV_{n}\right]}^{n+}\right)$$

The further reduction of the radical mono-cations to the fully uncharged viologen species DBV^0^ (labeled by P_3_)

(3) $${\left[DBV_{n}\right]}^{n+}+ne^{-}\Leftrightarrow nDBV^{0}$$

is not considered further here since this process occurs already within the regime of massive hydrogen evolution which prevents reliable STM measurements.

As argued in the introduction, it is expected that the respective charge state of the molecular species as well as the presence or absence of the anion underlayer will influence the structure of the adsorbed DBV layer. In the following we present and discuss STM images obtained under non-reactive conditions, *i.e.*, the molecules are adsorbed at a potential where they retain their dicationic character in the adsorbed state, as well as after a potential change leading to either the first reduction step to DBV^+•^ or to chloride desorption/adsorption, manifested by the peak pairs P_1_/P_1_′ and P_2_/P_2_′ in the CV, respectively.

#### 2.2.2. Structural Determination

**Figure 3:**
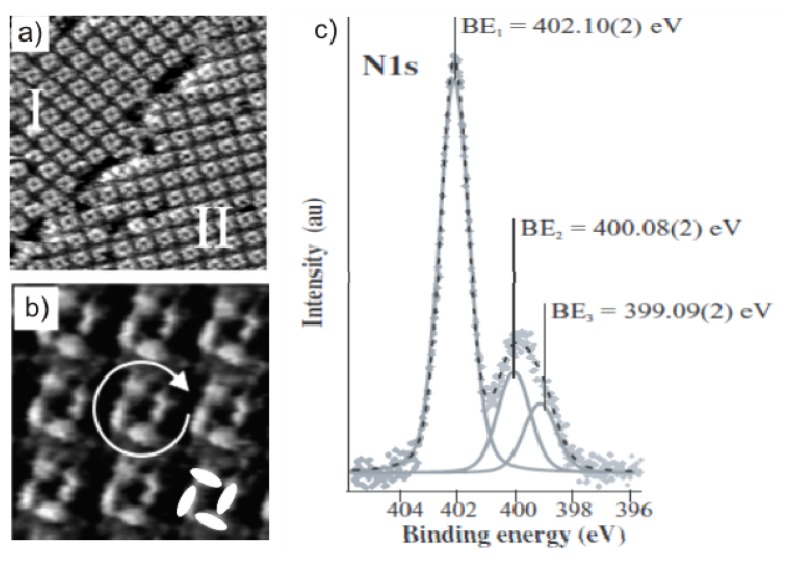
*In situ* STM images of the DBV^2+^ based “cavitand phase” forming on a *c*(2 × 2) – *Cl* covered Cu(100) surface: (**a**) 30 nm × 30 nm, *I*_t_ = 0.25 nA, *U*_b_ = 86 mV, *E* = + 70 mV; (**b**) 8.3 × 8.3 nm, *I*_t_ = 0.25 nA, *U*_b_ = 86 mV, *E* = +50 mV; (**c**) N1s photoemission spectrum of the DBV^2+^ monolayer phase on the *c*(2 × 2) – *Cl*/Cu(100) surface, *E*_photon_ = 720 eV; the copper sample was emersed at *E*_emers_ = +100 mV (see Figure 2) and transferred from the electrochemical environment into UHV. Reproduced with permission from [54–56].

**Figure 4:**
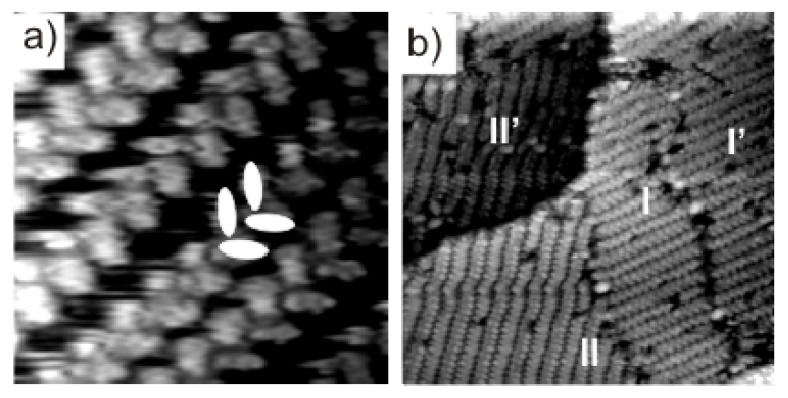
High resolution and large scale STM image of the DBV^2+^ herring-bone phase on *c*(*p* × √3) – *Cl*/Cu(111): (**a**) 8.5 nm × 8.5 nm: *I*_t_ = 0.1 nA, *U*_b_ = +386 mV, *E* = +10 mV; (**b**) 46.67 nm × 46.67 nm, *I*_t_ = 0.1 nA, *U*_b_ = +386 mV.

Exposing a *c*(2 × 2) – *Cl* and a *c*(*p* × √3) – *Cl* terminated Cu(100) and Cu(111) surface, respectively, to a DBV^2+^ containing electrolyte within the *non-reactive* double layer regime results in the instantaneous formation of a highly ordered DBV^2+^ film (Figures 3 and 4).

As seen in Figure 3 on Cl/Cu(100), a highly ordered layer of DBV^2+^ is observed at the electrode/electrolyte interface forming the well known “cavitand” phase [38,54–56]. Figure 3a shows two mirror domains of this phase denoted as I and II enclosing an angle of 31° ± 1° between each other. On the molecular level in Figure 3b, it becomes visible that one cavitand consists of four individual DBV^2+^ species forming a square-shaped motif enclosing a cavity in the center. Since two different arrangements of the four building blocks are possible (one of which is accentuate in Figure 3b), these cavitands occur in two circularly chiral enantiomers [55]. The dicationic character of the DBV^2+^ building blocks of this cavitand structure was verified by immersing the electrode out of the electrolyte at *E*_emers_ = +100 mV *vs*. RHE (see Figure 2) and measuring the N1s photoemission spectrum (Figure 3c). Three separate spectral components are observed with the dominant component at BE_1_ = 402.10(2) eV and two smaller ones at lower binding energies, *i.e*., BE_2_ = 400.08(2) eV and BE_3_ = 399.09(2) eV, respectively. These observed peaks are assigned to the respective three redox states of the adsorbed viologen species, namely the dicationic DBV^2+^, the mono-cationic DBV^•+^ and the uncharged species (DBV^0^) [56]. The appearance of reduced viologen species after emersion in this potential regime, where only DBV^2+^ should exist, is due to radiation damage, *i.e.*, to an instability of the di-cationic viologen species against the x-ray beam under UHV conditions. This interpretation is in agreement with Liu *et al.*[57] who investigated the N1s photoemission spectrum of 1,1′-bis(4-vinyl-benzyl)-viologen, in which the main component at the highest binding energy of BE_1_ = 401.7 eV was attributed to the positively charged nitrogen of the di-cationic viologen species (V^2+^). Two lower binding energy peaks, *i.e.*, at BE_2_ = 399.5 eV and BE_3_ = 398.6 eV were assigned to the corresponding mono-reduced viologen species (V^•+^) and the fully uncharged viologen (V^0^), whose intensities grow with irradiation time at the expense of the intensity at BE_1_ = 401.7 eV.

Using the *c*(*p* × √3) – *Cl*/Cu(111) surface as substrate for the viologen adsorption at potentials more positive than peak P_1_ in Figure 2b the di-cationic viologen species (DBV^2+^) adsorbs again strongly at the Cl/Cu(111)/electrolyte interface, however, forming a so called “herring bone” phase [58,59]. Figure 4 shows a large scale and a high resolution STM image of this DBV^2+^ herring bone structure. Unlike the dication phase on Cl/Cu(100) which consists of quadratic cavitands formed by four individual DBV^2+^ species (Figure 3), on the (nearly) threefold symmetric *c*(*p* × √3) – *Cl*/Cu(111) surface the DBV^2+^ molecules arrange in rows, and a building block of the herring bone phase consists of only two individual di-cationic viologen species which enclose an angle of 120° ± 1° between each other (Figure 4a). On the mesoscopic length scale these DBV^2+^ herring bones arrange themselves into extended domains which are again rotated by an angle of 120° ± 1° with respect to each other as observed in Figure 4b. The fact that both the quadratic molecular lattice on the *c*(2 × 2) – *Cl*/Cu(100) surface and the arrangement on the *c*(*p* × √3) – *Cl*/Cu(111) surface are dominated by angles of 90° and 120°, respectively, hints to a template effect rather than a mere self-assembly of the molecular dications on both surfaces.

**Figure 5:**
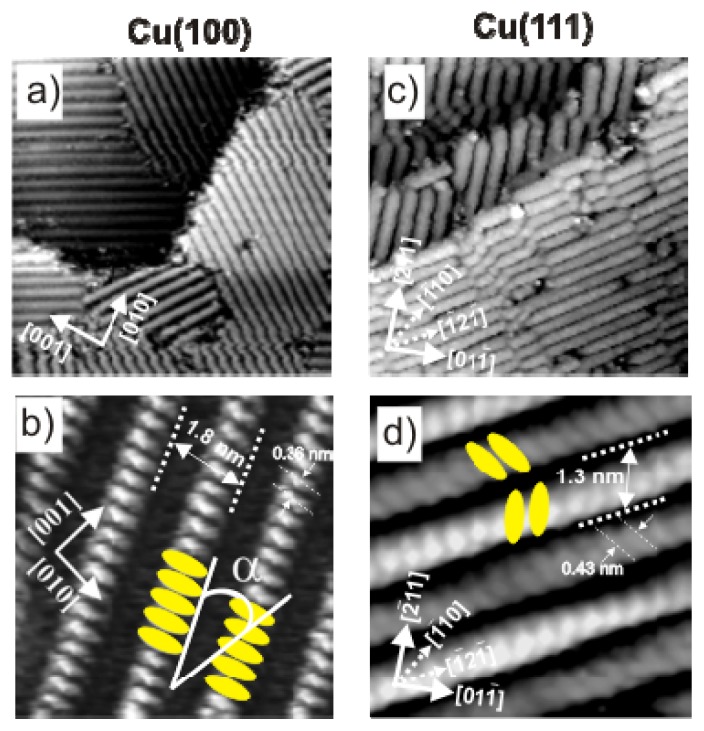
Surface morphologies and molecular arrangements on *c*(2 × 2) – *Cl*/Cu(100) and *c*(*p* × √3) – *Cl*/Cu(111) in the presence of DBV^•+^ radical monocations. “Parallel stacking” phase on Cu(100): (**a**) 44.37 nm × 44.37 nm, *I*_t_ = 0.1 nA, *U*_b_ = +350 mV, *E* = −300 mV; (**b**) 6.8 nm × 6.8 nm, *I*_t_ = 0.2 nA, *U*_b_ = +93 mV, *E* = −200 mV. “Alternating stacking” phase on Cu(111): (**c**) 35.49 nm × 35.49 nm, *I*_t_ = 0.1 nA, *U*_b_ = 386 mV, *E* = −280 mV; (**d**) 5.06 nm × 5.06 nm, *I*_t_ = 0.1 nA, *U*_b_ = 298 mV, *E* = −286 mV.

Sweeping the electrode potential negatively, *i.e.*, passing peak P_1_ in Figure 2a,b, both the cavitand and the herring-bone phase become desintegrated, and gradually completely new stripe patterns are formed as seen in Figure 5. These structure transitions are a consequence of the reduction of the viologen di-cationic species (DBV^2+^) to the corresponding viologen radical mono-cations (DBV^•+^) according to Equations 1 and 2. On both surfaces the stripes are formed by polymeric chains of π–π stacked DBV^•+^monocation radicals within which neighboring molecular species are arranged in a parallel face-to-face manner. The bright lengthy dots are assigned to the π-electron system of the bipyridinium moieties, while the benzyl groups are assumed to be located in the dark ditches between adjacent bipyridinium chains [38]. The distance between adjacent stripes is *s* = 1.8 nm and *s* = 1.3 nm on the Cl/Cu(100) and Cl/Cu(111) substrate, respectively, suggesting a stronger overlap between benzyl groups of adjacent stripes on the Cl/Cu(111) surface (Figure 5). Further distinct differences are observed in the internal molecular arrangement of the stripes on Cl/Cu(100) *vs.* Cl/Cu(111), respectively. While the intermolecular distance *within* the DBV^+•^ stripes on the *c*(2 × 2) – *Cl*/Cu(100) substrate is *d* = 3.6 ± 0.1 Å, this distance is *d* = 4.3 Å on the *c*(*p* × √3) – *Cl*/Cu(111) surface; both values being within the typical range of π–π stacked assemblies of aromatic systems [60]. Such values have also been observed for π–π stacked phases of 2,2′-bipyridine on Au(100) [61] and Au(111) [25].

Moreover, while the DBV^+•^ radical monocations in all stripes within one adsorbate domain on Cl/Cu(100) are oriented parallel (yellow dots in Figure 5b), the orientation of the radical monocations in adjacent stipes on the Cl/Cu(111) substrate is alternating, *i.e.*, the molecules are rotated by 120° with respect to each other (yellow dots in Figure 5d), leading to a zig-zag appearance. Furthermore, adjacent rows of the alternating stripe structure appear differently bright in the STM image. Considering the uniaxial incommensuracy of the chloride structure underneath the most likely explanation for this phenomenon is that molecules in adjacent rows are situated in inequivalent adsorption sites.

Obviously, the difference in the structure of the adsorbed viologen layers on the two kinds of surfaces is governed by the structural properties of the substrate *via* the chloride interlayer. A quadratic DBV^2+^ structure is formed on the Cl/Cu(100) electrode induced by the four-fold symmetry of the (1 × 1)-Cu(100) lattice. While the individual dicationic bipyridinium moieties are parallel to densely packed Cl-rows underneath [38], the rows of cavitands are not parallel to any commensurate directions of the *c*(2 × 2) – *Cl* phase resulting in the formation of mirror domains within the DBV^2+^ adlayer (Figure 3a). For symmetry reasons, there are four orientations of rotational domains in total on the whole Cu(100) surface.

By contrast, three orientations of DBV^2+^ herring-bone domains, rotated by 120°, are observed if the *c*(*p* × √3) – *Cl*/Cu(111) electrode is employed as substrate, hinting to the influence of the (quasi) hexagonal structure of the chloride modified Cu(111) substrate. Moreover, here the molecular rows run parallel to the step-edges, hence, parallel to the 〈 2̄ 11〉 directions of the Cu(111) substrate, suggesting that the electrostatic interaction between the molecules and the chloride precovered copper substrates is stronger on Cu(111) than on Cu(100).

**Figure 6:**
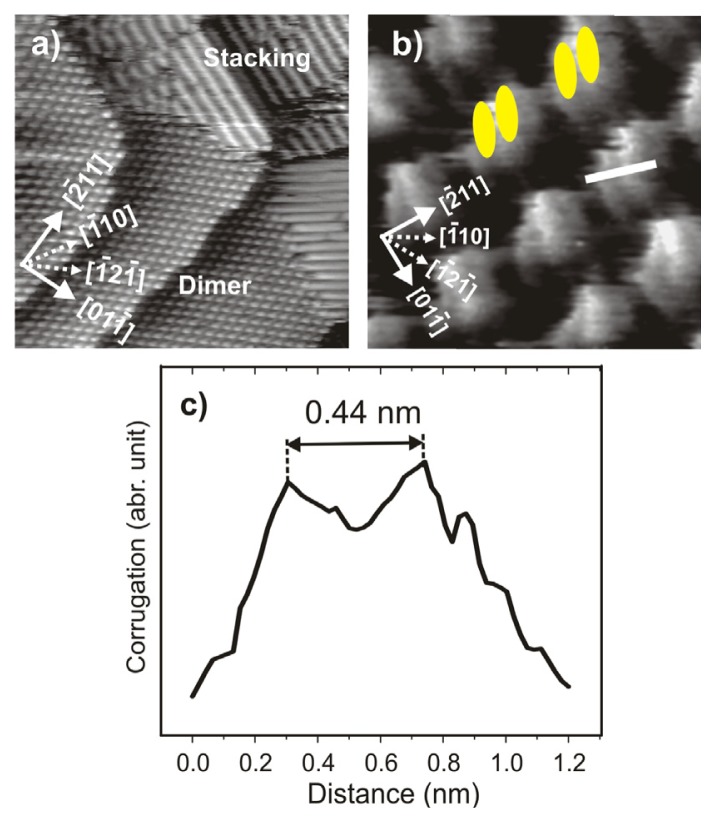
*In situ* STM images showing the co-existence of dimer phase and alternating stripe phase on the Cl/Cu(111) surface: (**a**) 46.67 nm × 46.67 nm, *U*_b_ = 175 mV, *I*_t_ = 0.2 nA, *E* = −310 mV; (**b**) Atomically resolved STM images of the dimer phase: 5.63 nm × 5.63 nm, *U*_b_ = 355 mV, *I*_t_ = 0.1 nA, *E* = −295 mV; (**c**) Line profile recorded along the white line in panel **b** indicating an intermolecular distance within a dimer of 0.44 nm.

Regarding the findings presented later in section 2.4 we mention here that the reduction induced phase transitions of DBV^2+^ on Cl/Cu(100) and Cl/Cu(111) may be accompanied by so-called “dimer-phases”. However, while on the Cl/Cu(100) surface such dimer-phase occurs only if the DBV^2+^ dications are absorbed under “reactive conditions”, *i.e*., in the potential regime between −120 mV and +250 mV *vs.* RHE, where sticking on and reduction at the surface take place in parallel, on the Cl/Cu(111) surface a dimer-phase is also formed from stable DBV^2+^ surface species, *i.e.*, adsorbed DBV^2+^ dications forming the herring-bone structure at positive potentials. The dimer-phase on Cl/Cu(100) transforms irreversibly into either the stripe- or the cavitand-structure when going to negative or positive potentials, respectively [38], indicating its metastability. In turn, the dimer-phase on Cl/Cu(111) coexists with the “alternating stripe” phase (see Figure 6a), both forming reversibly out of the herring-bone phase. Close-packed rows of the dimer-phase are also aligned along the step-edges like the chains of monocation-radicals in the alternating stripe phase. The high resolution STM image in Figure 6b reveals further details of the internal molecular structure, *i.e.*, the orientation and packing arrangement in the ordered dimer adlayer. Individual spots are recognized as double-rodlets with an inner spacing of 0.44 ± 0.02 nm, as deduced from the line profile in Figure 6c, typical for π–π stacked aromatic systems [60]. The orientation of the two rodlets encloses the same angle of 120° (60°) with the direction of the dimer rows as the monocation-radicals do with the direction of the alternating stripes, with the exception that the molecular orientation of the dimers does not alternate from dimer-row to dimer-row. Thus, the dimer-phase on the Cl/Cu(111) surface appears to be a precursor state in the formation process of the “alternating stripe” phase.

In addition, a chloride desorption/re-adsorption induced order/disorder phase transition which is marked by the P_2_/P_2_′ peak pair in Figure 2 is also observed in both cases. Details are presented and discussed by Pham *et al.* in [54] and a forthcoming paper [58].

### 2.3. H_2_TTMAPP Adlayers on Chloride Modified Cu(100) and Cu(111)

**Figure 7:**
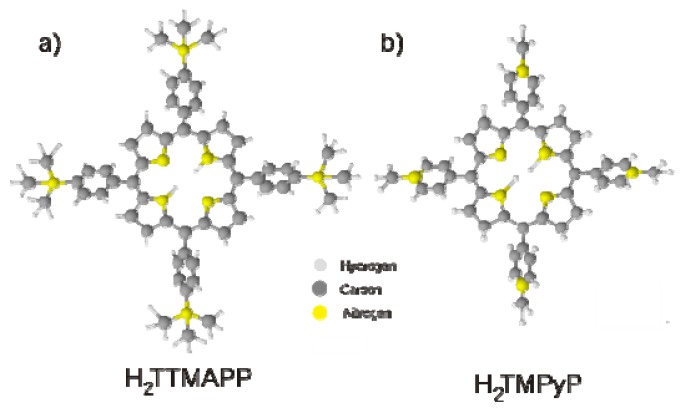
The molecular structures of 5,10,15,20-Tetrakis (4-trimethyl ammonium phenyl) porphyrin tetra p-toluenesulfonate) and 5,10,15,20-Tetrakis-(*N*-methyl-4-pyridyl)-21H,23H-porphyrin tetratosylate) abbreviated as H_2_TTMAPP (**a**) and H_2_TMPyP (**b**), respectively.

In this section the electrochemical behavior and the structural features of a layer of 5,10,15,20-Tetrakis (4-trimethyl ammonium phenyl) porphyrin tetra (p-toluenesulfonate) molecules (abbreviated as H_2_TTMAPP), in which the four hydrogen atoms at the four meso positions of the central porphyrin core are replaced by four trimethy-ammonium-phenyl groups (Figure 7), are presented and discussed in detail.

#### 2.3.1. Electrochemical Behavior

**Figure 8:**
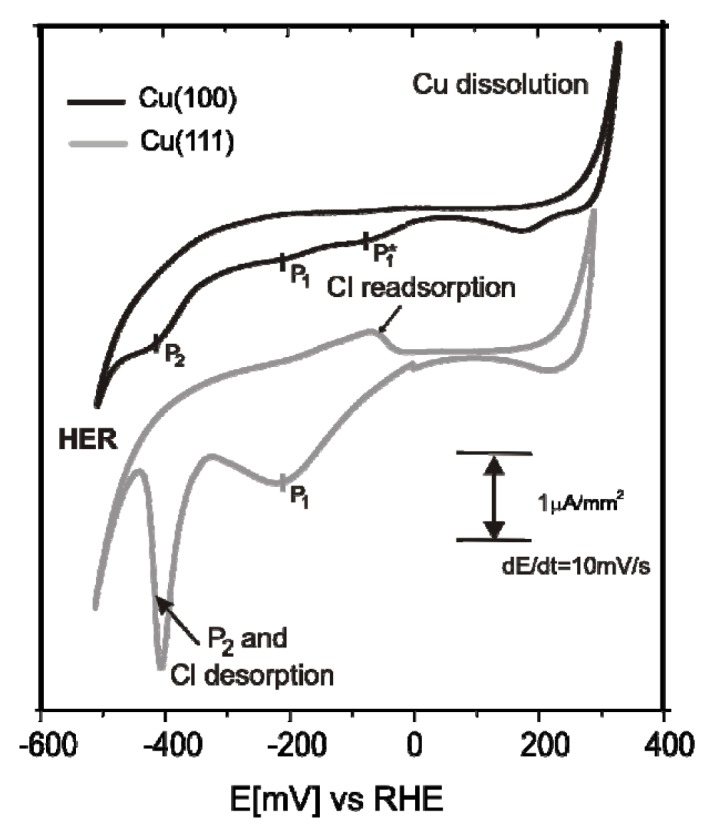
Cyclic voltammograms of Cu(100) (upper black curve) and Cu(111) (lower grey curve) electrodes in an electrolyte containing [H_2_TTMAPP]^4+^ cations (10 mM HCl + 0.1 mM H_2_TTMAPP). P_1_/P_1_* and P_2_ indicate the stepwise reduction of the H_2_TTMAPP molecules, d*E*/d*t* = 10 mV/s.

Figure 8 shows typical cyclic voltammogams (CV) of Cu(100) and Cu(111) electrodes in HCl acid solution containing [H_2_TTMAPP]^4+^ cations (10 mM HCl + 0.1 mM H_2_TTMAPP). Note, the CVs of both Cu(100) and Cu(111) electrodes in pure supporting chloride solution (10 mM HCl) are shown in Figure 2 and have been discussed in section 2.1 and in great detail in [49,53] and papers cited therein. The CV of the Cu(100) electrode in the [H_2_TTMAPP]^4+^ containing electrolyte is depicted by the upper black curve in Figure 8. Within the potential window limited by the anodic copper dissolution reaction (CDR) and the cathodic hydrogen evolution reaction (HER), two pronounced cathodic peaks, P_1_ at *E*_P1_= −210 mV enclosing a small shoulder P_1_* at *E*_P1*_ = −80 mV, and peak P_2_ at *E*_P2_ = −420 mV *vs.* RHE, appear, indicating the stepwise reduction of the molecules [62].

Similarly, the lower grey CV in Figure 8 shows the electrochemical behavior of the Cu(111) electrode in the [H_2_TTMAPP]^4+^ cations containing electrolyte. Besides the appearance of the chloride desorption and adsorption peaks at *E* = −400 mV and *E* = −100 mV, respectively (see also Figure 2), the curve is dominated by a broad cathodic peak P_1_ at *E*_P1_= −210 mV which may very well be a superposition of P_1_ and P_1_* seen in the upper CV of the Cu(100) electrode. Note, that the corresponding re-oxidation peaks are out of the potential window of the copper electrodes, *i.e.*, beyond the CDR. These peaks are only observed when HOPG serves as the working electrode; for details see [62].

Based on the observed results and the chemical structure of the employed [H_2_TTMAPP]^4+^ molecules, we assign the first pair of peaks P_1_/P_1_′ to the first reduction step of the free base porphyrin [H_2_TTMAPP]^4+^ arising from the first two-electron transfer process [62].

(4) $${\left[{\text{H}}_{2}\text{TTMAPP}(0)\right]}^{4+}+2{\text{e}}^{-}+2{\text{H}}^{+}↔{\left[{\text{H}}_{4}\text{TTMAPP}(-\text{II})\right]}^{4+}$$

The second reduction peak P_2_ has been proposed to correspond to a four-electron transfer step [63–65].

(5) $${\left[{\text{H}}_{4}\text{TTMAPP}(-\text{II})\right]}^{4+}+4{\text{H}}^{+}+4{\text{e}}^{-}\to {\left[{\text{H}}_{8}\text{TTMAPP}(-\text{VI})\right]}^{4+}$$

In the following the specific structural features as well as structural phase transitions of the adsorbed porphyrin adlayers on both, *c*(2 × 2)*Cl*-Cu(100) and *c*(*p* × √3)*Cl*-Cu(111), taking place upon reaching the first reduction peak P_1_ in the respective CV, are presented and discussed.

#### 2.3.2. Structural Characterization

Exposure of both chloride modified copper surfaces, Cu(100) and Cu(111), to the [H_2_TTMAPP]^4+^ containing electrolyte within the double layer regime above P_1_ in the respective CV (Figure 8) results in an instantaneous adsorption of a laterally highly ordered layer of [H_2_TTMAPP]^4+^ species at the copper/electrolyte interface.

**Figure 9:**
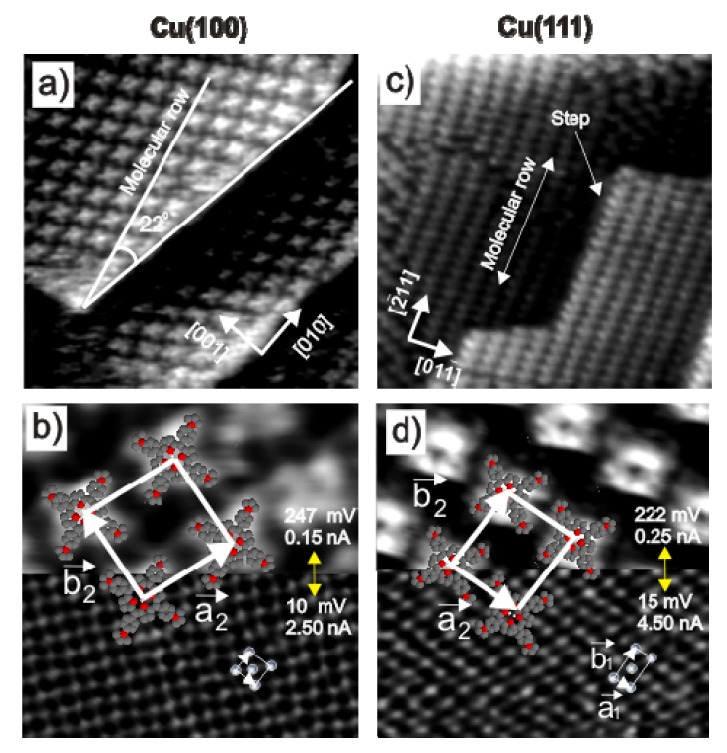
Typical medium and high resolution in situ STM images of [H_2_TTMAPP]^4+^ adlayers formed on chloride precovered Cu(100) and Cu(111) electrode surfaces. (Small deviations from the characteristic angle of 90° (120°) between step edges on the Cu(100) (Cu(111)) surface are due to slight drift effects.) A stronger electrostatic interaction between the porphyrin overlayer and the underlying chloride lattice is suggested by the directional coincidence between molecular rows and step edges on the Cl/Cu(111) substrate. (**a**) 24.42 nm × 24.42 nm, *I*_t_ = 0.1 nA, *U*_b_ = 168 mV, *E* = +20 mV; (**b**) Upper and lower half: 7.90 nm × 3.95 nm, *E* = −20 mV; (**c**) 33.46 nm × 33.46 nm, *I*_t_ = 0.1 nA, *U*_b_ = 346 mV, *E* = +50 mV; (**d**) Upper and lower half: 8.10 nm × 4.05 nm, *E* = +20 mV.

Figure 9a,b depict the surface morphology and molecular structure of the [H_2_TTMAPP]^4+^ overlayer forming on the *c*(2 × 2) – *Cl*/Cu(100) surface. First of all the straight step-edges in Figure 9a running in orthogonal directions prove the persistence of the *c*(2 × 2) – *Cl* layer underneath the organic molecules. This indicates that the [H_2_TTMAPP]^4+^ adlayer has no significant impact on the surface morphology which is governed by the chemisorptive Cu–Cl bond. On top of this chloride layer the [H_2_TTMAPP]^4+^ species are arranged in rows. The direction of the molecular rows forms an angle of 22° ± 1° with the direction of the steps, *i.e.*, with a [001] direction of the copper substrate. The molecular rows, hence, neither run parallel to any high symmetry axes of the chloride lattice underneath nor to the close-packed atom rows of the Cu(100) substrate. By reason of surface symmetry, therefore, there are two co-existing mirror domains with respect to each close-packed direction of the chloride mesh underneath. Consequently, by taking the quadratic symmetry of the chloride lattice into account, the adsorbed porphyrin species self-arrange into four possible rotational domains in total on the chloride terminated Cu(100) electrode surface.

A spontaneously ordered adlayer forms also on the *c*(*p* × √3) – *Cl*/Cu(111) as shown in Figure 9c,d. After [H_2_TTMAPP]^4+^ adsorption the typical chloride induced angle of 120° between step-edges still remains. Thus, the presence of the molecules on the surface has again no impact on the typical morphology of the chloride precovered substrate. However, unlike on the Cl/Cu(100) surface the close-packed molecular rows run parallel to step-edges which are aligned along the close-packed chloride rows underneath [53]. As a result, the [H_2_TTMAPP]^4+^ molecular rows are oriented parallel to a high symmetry direction of the chloride lattice. Alternatively, they are aligned parallel to the 〈2̄11〉 directions of the Cu(111) substrate. This observation indicates that the electrostatic interaction between the [H_2_TTMAPP]^4+^ overlayer and the *c*(*p* × √3) – *Cl*/Cu(111) lattice underneath appears to be stronger than that between the [H_2_TTMAPP]^4+^ adlayer and an underlying *c*(2 × 2) – *Cl*/Cu(100) surface. This hypothesis is further supported by the molecular densities calculated further below.

High resolution STM images reveal a quadratic lattice (Figure 9b) of the [H_2_TTMAPP]^4+^ adlayer on the underlying *c*(2 × 2) – *Cl*/Cu(100) surface, while a rectangular [H_2_TTMAPP]^4+^ pattern is formed on the *c*(*p* × √3) – *Cl*/Cu(111) substrate as imaged in Figure 9d. Individual [H_2_TTMAPP]^4+^ molecules are recognized as a four-fold propoller with a hole in their center [62]. This indicates that these metal-free porphyrin molecules are lying flat on the Cl/Cu(100) and Cl/Cu(111) surfaces as illustrated by the superimposed molecular models in Figure 9b,d. This orientation of the molecules is expected because it optimizes the interaction between their large planar π-system and the substrate [14].

Applying different tunneling conditions a more detailed structural correlation between the [H_2_TTMAPP]^4+^ overlayer structure and the underlying chloride lattice can be worked out. Indeed, the first and second half of the STM images displayed in Figure 9b,d are recorded at the same surface area but monitored under different tunneling conditions. Under “moderate” tunneling conditions, *i.e.*, with high bias voltage and low tunneling current, the tunneling tip is further away from the surface and, hence, the covering porphyrin adlayer is imaged as shown in the upper halves in Figure 9b,d. By contrast, under more “drastic” tunneling conditions, *i.e.*, with low bias voltage and high tunneling current, the tunneling tip is brought close to the electrode surface and acts as “molecular brush” which locally sweeps the porphyrin molecules away upon scanning, leaving the underlying chloride lattice behind (lower halves in Figure 9b,d). Obviously, on the Cl/Cu(100) electrode the molecular rows run parallel neither to the close-packed anion rows corresponding to the [001] direction of the chloride underlayer nor, hence, to the close-packed Cu atom rows of the Cu(100) electrode. By extrapolation of the upper half STM image onto the lower half in Figure 9b it becomes evident that the direction of the molecular rows is rotated by an angle of 23° ± 1° with respect to the [001] direction of the chloride lattice. As a consequence the angle between the close-packed direction of porphyrin rows and the [010] direction of the copper lattice is 22° ± 1°. The quadratic unit cell as marked in Figure 9b contains one molecule and can be described either by a $\left|\begin{array}{ll} 5 & 2\\ -2 & 5 \end{array}\right|$ transformation matrix or by a $\left(\sqrt{29}\times \sqrt{29}\right)R23^{0}$ structure with respect to the *c*(2 × 2) – *Cl* lattice serving as the internal calibration lattice. Consequently, the lattice vectors of the molecular mesh are calculated to be |*a⃗*_2_| = |*b⃗*_2_*|* = 1.95 ± 0.1 nm, enclosing an angle of 91° ± 2°. On the basis of this result the surface coverage per domain of the porphyrin adlayer is calculated to be 0.0345 ML relative to the density of the chloride layer, or 2.63 × 10^13^ molecules/cm^2^[62].

In contrast to the molecular arrangement on Cu(100) the orientation of the molecular [H_2_TTMAPP]^4+^ rows adsorbed on the *c*(*p* × √3) – *Cl*/Cu(111) surface run parallel to the close-packed rows of chloride anions underneath (Figure 9d) which corresponds to the 〈2̄11〉 directions of the Cu(111) substrate [53]. Analogous to the molecular adsorption on the Cu(100) electrode, a superposition of the two consecutive half STM images in Fig. 8d taken with different tunneling conditions to show either the molecular overlayer or the chloride underlayer reveals a correlation between the [H_2_TMAPP]^4+^ structure and the chloride lattice underneath. As a result, the unit cell (*a⃗*_2,_*b⃗*_2_) drawn in Figure 9d contains one porphyrin molecule and may be described by a (3 × 4) superstructure with respect to the *c*(*p* × √3) – *Cl* layer underneath. The lattice vectors, therefore, are calculated to be |*a⃗*_2_| = 1.75 ± 0.1 nm and |*b⃗*_2_| = 1.95 ± 0.1 nm, respectively, enclosing an angle of 90° ± 2°. The surface coverage per domain is calculated to be 0.042 ML with respect to the chloride underlayer, or 2.8 × 10^13^ molecules/cm^2^. This value is bigger than that on the chloride modified Cu(100) substrate which is in line with stronger electrostatic interaction between the [H_2_TTMAPP]^4+^ cations and the higher density of chloride anions on Cu(111).

As mentioned in the electrochemistry section above, the [H_2_TTMAPP]^4+^ cations undergo two successive reduction steps taking place within the potential window of the copper electrodes (see Equations 4 and 5). As stated before, it is reasonable to expect that this change in charge state may also lead to a change in adsorbate structure. In the following this is exemplarily presented and discussed for the first reduction step on the Cl/Cu(111) electrode surface.

**Figure 10:**
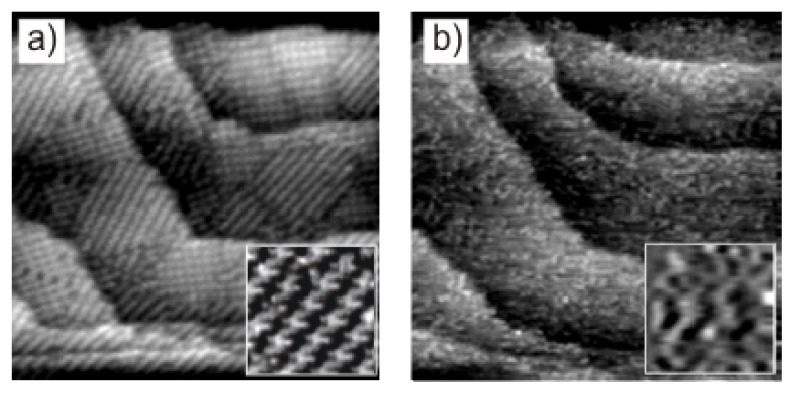
Molecular order/disorder phase transition on Cl/Cu(111) caused by the first two-electron [H_2_TTMAPP]^4+^ reduction: 76.82 nm × 76.82 nm and inset: 6.46 nm × 6.46 nm; (**a**) *I*_t_ = 0.1 nA, *U*_b_ = +274 mV, *E* = +10 mV; (**b**) *I*_t_ = 0.1 nA, *U*_b_ = +276 mV, *E* = −240 mV.

Figure 10 shows two consecutive STM images recorded at the same surface area while the electrode potential is swept from the right to the left side of the peak P_1_ appearing in the CV of Cu(111) in Figure 8. Obviously, an order/disorder phase transition takes place on the surface when the [H_2_TTMAPP]^4+^ cations undergo the first reduction step. While a fully ordered monolayer of the porphyrin molecules is observed at a potential of *E* = +10 mV (Figure 10a and inset) a disordered layer is observed on the Cl/Cu(111) surface at *E* = −240 mV as depicted in Figure 10b and the inset. This finding agrees with the expectation that the reduction of the molecules will modify the delicate balance between the substrate-adsorbate and adsorbate-adsorbate interactions. This reduction of the free base porphyrin from the initial state ([H_2_TTMAPP(0)]^4+^) to the corresponding reduced product, *i.e.*, [H_4_TTMAPP(-II)]^4+^, according to Equation 4, leaves the molecules less positive. Both the attractive electrostatic interaction between the positively charged [H_2_TTMAPP]^4+^ species and the adsorbed chloride anions on the copper electrode surface as well as the mutual repulsive electrostatic interaction between neighboring molecules is significantly reduced when the [H_2_TTMAPP]^4+^ cations undergo this first reduction step. As a consequence, the decay of the previous order is detected.

### 2.4. Bimolecular Porphyrin/Viologen Adlayer on Chloride Modified Cu(111) and Cu(100)

As mentioned in the previous sections, in electrochemical environment [H_2_TTMAPP(0)]^4+^ cations undergo two consecutive redox reactions involving six electrons in total (Equations 4 and 5). In turn, as described in section 2.2 there are three possible redox-states of adsorbed DBV, namely, the dications DBV^2+^, the radical mono-cations DBV^•+^ and the uncharged DBV^0^ molecules, which transform into each other *via* two single-electron transfer steps. In the following we present results for the competitive adsorption of the two positively charged organic species, namely porphyrin ([H_2_TTMAPP]^4+^) and dibenzyl-viologen (DBV^2+^) on a Cu(100) and a Cu(111) surface, respectively, using again the combination of cyclic voltammetry and *in situ* STM.

#### 2.4.1. Electrochemical Behavior

**Figure 11:**
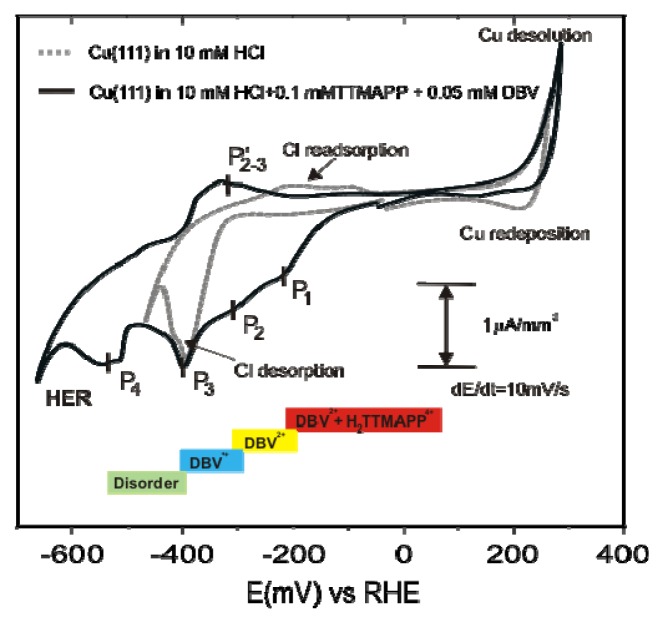
Cyclic voltammogram of a Cu(111) electrode in pure hydrochloric acid (dotted grey curve) and in an electrolyte containing a mixture of [H_2_TTMAPP]^4+^ and DBV^2+^ cations (black curve); the four cathodic peaks P_1_–P_4_ relate to both the stepwise reduction of the porphyrin- and the viologen-cations; scan rate d*E*/d*t* = 10 mV/s. The bars below the CVs indicate the existence regime of the respective ordered species or disorder.

Representative steady-state cyclic voltammograms of a Cu(111) surface in both the supporting HCl-solution (10 mM HCl) (dotted grey curve) and the electrolyte containing a mixture of both organic species (10 mM HCl + 0.1 mM TTMAPP + 0.5 mM DBV) (black curve) are presented in Figure 11.

Compared to the CV in pure HCl a drastic change is observed in the mixed electrolyte right after the Cu(111) is brought into contact with the solution containing both organic species. The most striking deviation from the CV in the pure supporting electrolyte is seen in the cathodic potential regime where several characteristic features appear at P_1_= −193 mV, P_2_ = −301 mV, P_3_ = −398 mV and P_4_ = −527 mV *vs*. RHE, respectively. In addition, the HER is shifted to more negative potentials by 125 mV. Obviously, an organic overlayer is still present on the electrode surface in the HER potential regime, causing a hindrance of the HER. Based on the interpretation of the CVs of Cu(111) in the monomolecular solutions (Figures 2 and 8) the four distinguished cathodic peaks P_1_ to P_4_ in the black CV are attributed to (i) the first two-electron reduction step of the porphyrin cations; (ii) the first reduction process from the di-cationic DBV^2+^ viologen to the corresponding radical mono-cation DBV^•+^; (iii) the chloride desorption, and (iv) the reduction of the viologen radical mono-cations to the fully uncharged viologen species (DBV^0^), respectively.

A very similar electrochemical behavior is also observed with a Cu(100) electrode surface brought into contact with the same mixed electrolyte, and is therefore not explicitly shown here [59,66].

#### 2.4.2. Structural Characterization

**Figure 12:**
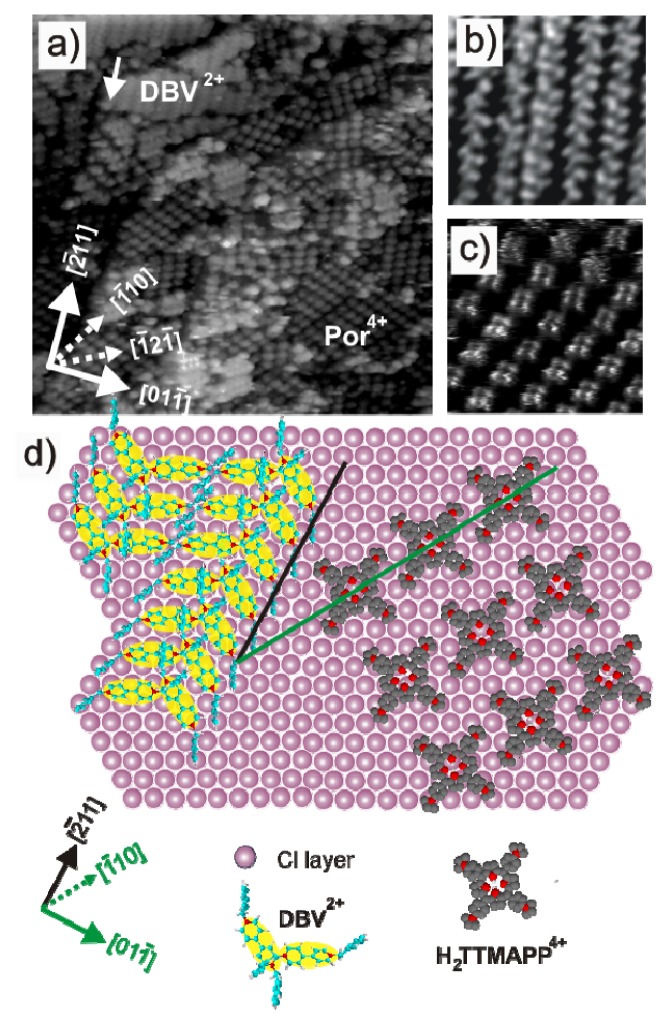
(**a**) Typical large scale in situ STM image showing co-existing domains of the [H_2_TTMAPP]^4+^ and the DBV^2+^ cations: 63.23 nm × 63.23 nm, *U*_b_ = 339 mV, *I*_t_ = 0.15 nA, *E* = −100 mV; and (**b**,**c**) high resolution STM images showing the herring-bone phase of DBV^2+^ and the square lattice formed by [H_2_TTMAPP]^4+^ species: (**b**) 9.46 nm × 9.46 nm; *U*_b_ = 285 mV, *I*_t_ = 0.2 nA, *E* = −100 mV (**c**) 7.2 nm × 7.2 nm; *U*_b_ = 240 mV, *I*_t_ = 0.3 nA, *E* = −100 mV; (**d**) Possible structure model for the co-existence of DBV^2+^ herring-bone and square [H_2_TTMAPP]^4+^ phase on the Cl/Cu(111) substrate.

Figure 12a shows a typical large scale in situ STM image of the Cu(111) surface recorded at *E* = −100 mV right after the pure supporting electrolyte (10 m M HCl) was replaced by one containing the mixture of the [H_2_TTMAPP]^4+^ and DBV^2+^ species. It can be seen that the terrace is fully covered with two randomly distributed structures, namely, a herring-bone pattern (Figure 12b) and a square shaped lattice (Figure 12c). On the basis of the STM results presented in the previous sections, it is evident that the former structure must be assigned to the adsorption of the DBV^2+^ dications [58,59], while the [H_2_TTMAPP]^4+^ molecules form the square lattice [62].

A careful analysis reveals that the DBV^2+^ cation rows run parallel to the substrate steps as marked by the white arrow in Figure 12a, whereas the rows of the [H_2_TTMAPP]^4+^ species are aligned along the √3 direction of the chloride lattice. Like in the case of a solution containing viologen only, the DBV^2+^ rows are parallel to the close packed rows of the underlying chloride lattice and, hence, are coincident with the 〈2̄11〉 directions of the copper substrate. In contrast, by alignment along the √3 direction compared to the step-edges the [H_2_TTMAPP]^4+^ molecular rows run parallel to the close packed rows of the underlying copper lattice, *i.e.*, the 〈1̄10〉 directions [53], as depicted in the structure model in Figure 12d.

**Figure 13:**
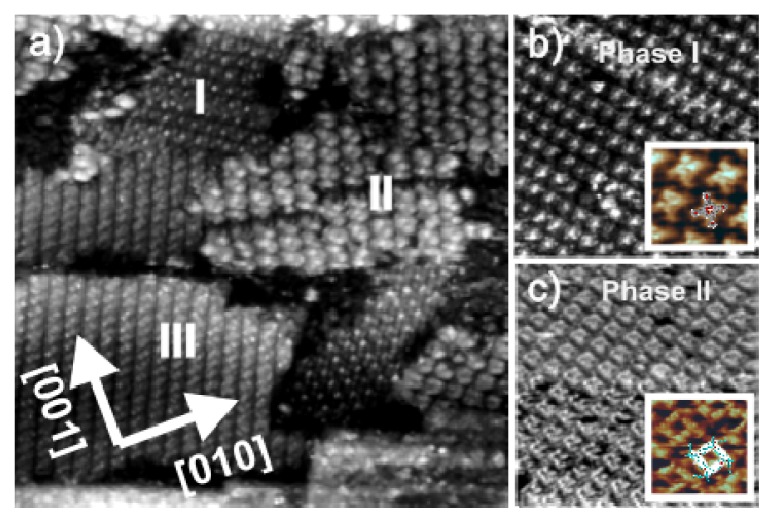
(**a**) Large scale in situ STM image illustrating three distinct phases forming on the Cl/Cu(100) electode surface: 61.46 nm × 61.46 nm, *U*_b_ = +363 mV, *I*_t_ = 0.2 nA, *E* = +10 mV; (**b**) Typical square phase of the [H_2_TTMAPP]^4+^ molecules on the Cl/Cu(100) electrode surface (phase I): 23.23 nm × 23.23 nm, *U*_b_ = +252 mV, *I*_t_ = 0.15 nA, *E* = +10 mV; Inset image: 4.95 nm × 4.95 nm, *U*_b_ = +168 mV, *I*_t_ = 0.1 nA, *E* = +20 mV; (**c**) Characteristic medium scale and high resolution STM images of a DBV^2+^ related cavitand phase (phase II): 30.37 nm × 30.37 nm, *U*_b_ = +355 mV, *I*_t_ = 0.1 nA, *E* = +0 mV.

Using Cl/Cu(100) as substrate for the bimolecular adsorption, again two phases can be distinguished formed by the respective [H_2_TTMAPP]^4+^ and DBV^2+^ adsorbates. Figure 13a shows a (nearly) square shaped phase I in which individual molecules are recognized as propellers (see Figure 13b and inset figure). Coexistent with this familiar [H_2_TTMAPP]^4+^ based square pattern the well known cavitand phase II built up by units of four dicationic DBV^2+^ viologen species is also observed (Figure 13c and inset). Both phases have already been characterized in detail in sections 2.2 and 2.3.

**Figure 14:**
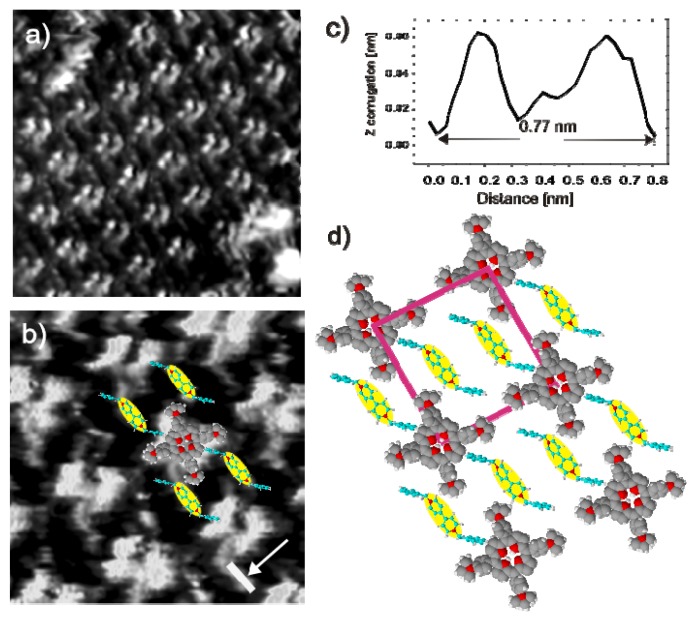
Typical medium scale and high resolution STM images of a mixed phase in which the [H_2_TTMAPP]^4+^ molecules are recognized as bright squares and the DBV^2+^ molecules as rodlets: *U*_b_ = +323 mV, *I*_t_ = 0.1 nA, *E* = +30 mV; (**a**) 12.82 nm × 12.82 nm; (**b**) 5.60 nm × 5.60 nm; (**c**) Line profile along the white bar in panel **b** (indicated by the arrow) representing the length of the bipyridinium core of a DBV cation; (**d**) Possible model for the mixed phase on Cl/Cu(100).

In addition to these two well known separated phases, a new phase made up by both porphyrin and viologen molecules intermixed is also observed (phase III). Figure 14 displays an STM image, in which this combined arrangement of [H_2_TTMAPP]^4+^ and DBV^2+^ molecules on the chloride terminated Cu(100) electrode can be seen (Figure 14a). A closer view at sub-molecular resolution (Figure 14b) reveals a homogeneously alternating arrangement of [H_2_TTMAPP]^4+^ and DBV^2+^ molecules in which the [H_2_TTMAPP]^4+^ molecules appear as propeller shaped moieties and the DBV^2+^ molecules as rodlets in between (Figure 14b). The length of the visible rodlets is measured to be about 7.7 ± 0.2 Å (Figure 14c), which differs only slightly from the theoretical N–N distance of a bipyridinium unit of 7.1 Å. A molecular arrangement of the mixed phase is proposed by the model shown in Figure 14d.

In this mixed phase the spaces between the [H_2_TTMAPP]^4+^ porphyrin cations are large enough so that the DBV^2+^ molecules can be accommodated within these spaces allowing the formation of the new binary phase. This sterical condition plus the electrostatic interaction between the positively charged [H_2_TTMAPP]^4+^ and the DBV^2+^ molecules and the negatively charged chloride lattice are assumed to be responsible for the stabilization of this phase on the chloride pre-covered Cu(100) electrode surface. It should be mentioned that such a mixed porphyrin/viologen phase could neither be found with [H_2_TTMAPP]^4+^ and DBV^2+^ on Cl/Cu(111), because there the rectangular unit cell offers less free space for DBV^2+^ coadsorption, nor on Cl/Cu(100) with TMPyP^4+^ (without the spacious *tri*-methylammonium-phenyl ligands as seen in Figure 7b) and DBV^2+^ in the solution, because the space between TMPyP^4+^ species is significantly smaller [67].

#### 2.4.3. Electron transfer Governed Phase Transition

Like in the monomolecular adlayers redox-induced phase transitions are also observed on both Cl/Cu(100) and Cl/Cu(111) electrode surfaces covered with bimolecular layers of [H_2_TTMAPP(0)]^4+^ and dicationic DBV^2+^ species.

**Figure 15:**
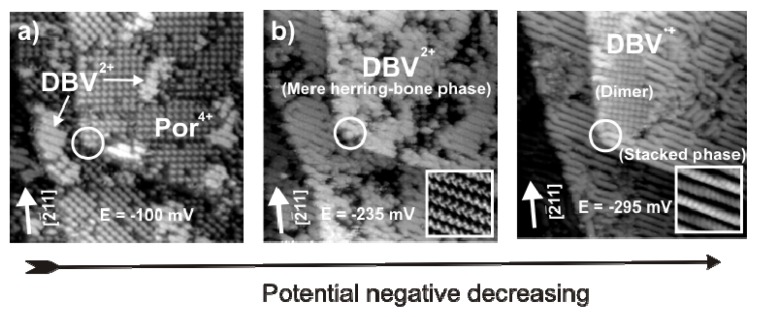
Potential driven phase transitions of H_2_TTMAPP^4+^ and DBV^2+^ domains on Cl/Cu(111) by passing the peaks P_1_ and P_2_ in Figure 11 in negative direction: 63.23 nm × 63.23 nm, *U*_b_ = 339 mV, *I*_t_ = 0.15 nA.

Figure 15 shows a series of STM images recorded at the same surface area (white circles mark the same surface position) for decreasing electrode potential passing the peaks P_1_ and P_2_ in the CV of Figure 11 in negative direction.

Figure 15a shows the coexistence of stable domains of the square phase of [H_2_TTMAPP(0)]^4+^ cations, as described in section 2.3, and of the DBV^2+^ herring-bone structure, as presented in section 2.2, at a potential of *E* = −150 mV. This structure breaks down once the potential is swept negatively across peak P_1_ in the CV of Figure 11. As seen in Figure 15b the whole surface becomes covered only by domains of the DBV^2+^ herring-bone structure. As described in section 2.3, in this potential regime the [H_2_TTMAPP(0)]^4+^ cations undergo a two-electron reduction step to the corresponding [H_4_TTMAPP(-II)]^4+^ species which desorbs. As a result, more DBV^2+^ dications can adsorb from the solution and finally occupy the whole surface in the form of the herring-bone structure, which is still stable at this potential (Figure 15b and inset).

This DBV^2+^ herring-bone structure on Cl/Cu(111) remains stable as long as the potential remains more positive than *E* = −240 mV *vs.* RHE. By sweeping toward even more negative potentials and passing peak P_2_ the herring-bone structure desintegrates and the growth of the corresponding stripe pattern is observed due to the reduction of the dicationic DBV^2+^ species to the corresponding radical mono-cationic DBV^•+^ species (Figure 15c) as described in section 2.2 [38,54]. The growth of this stacking phase preferentially occurs at defect points, domain boundaries and step-edges [61], and proves this structure to be the most stable phase at E = −285 mV. From a detailed analysis of the STM images, it is found that the dimer phase (Figure 6) accompanies the formation of the stripe pattern as marked in Figure 15c at the same potentials as in a solution containing only viologen [58,59].

**Figure 16:**
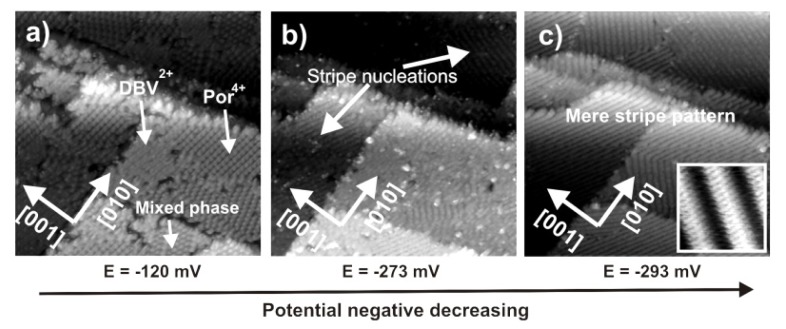
Series of STM images showing the surface transition from the mixed adlayer to the corresponding stripe pattern on the Cl/Cu(100) surface: 76.82 nm × 76.82 nm, *U*_b_ = +336 mV, *I*_t_ = 0.1 nA.

A similar behavior is also observed when the Cl-covered Cu(100) electrode serves as a substrate (Figure 16). In this case also the bimolecular adlayer comprising DBV^2+^ cavitand phase, square [H_2_TTMAPP]^4+^ phase as well as the mixed phase is desintegrated when the electrode potential is swept to negative potentials finally resulting in the formation of the DBV^*•^ stripe pattern only. The decay process starts at defect sites and/or domain boundaries marked by white arrows in Figure 16b, which serve as “active centers” for the phase transition taking place on the copper surface.

The stripe phases on both the Cl/Cu(111) and Cl/Cu(100) surface exhibit the same structural characteristics as in the respective monomolecular adlayers (see sections 2.2 and 2.3). The binary molecular structure can be restored on both copper substrates by sweeping the working potential back to positive potentials because the first electron transfer steps occurring with respect to porphyrin and viologen molecules are reversible processes.

## 3. Experimental Section

H_2_TTMAPP and DBV were purchased from Sigma-Aldrich, hydrochloric acid was ordered from Merck and used without further purification.

The Cu(100) and Cu(111) single crystal used in this experiments were manufactured by MaTech Co. (Juelich, Germany). Prior to the STM measurement, the copper samples must be electropolished in order to remove the massive oxide film from the copper surface formed in air by immersing the copper surface into 50% orthophophiric acid at an anodic potential of 2 V for about 20–40 s. To guarantee a reproducibly smooth surface even after several electropolishing procedures, a surface orientation of the copper single crystals of less than 0.5° off the (100) and (111) planes is required.

For all solutions high purity water (Milli-Q purification system, conductivity >18 MΩ cm, TOC< 4 ppb) and other reagent grade chemicals were used. All electrolyte solutions were purged with oxygen free argon gas for several hours before use. The potentials of the copper electrodes are quoted with respect to a reversible hydrogen electrode (RHE) while a Pt wire is employed as counter-electrode.

The tunneling tips used in our experiments were electrochemically etched from 0.25 mm tungsten wire in 2 mM KOH solution and subsequently isolated by passing the tip through a hot melt glue film in order to minimize residual Faradaic currents.

All STM measurements presented in this contribution were carried out using a home-built electrochemical scanning tunneling microscopy (EC-STM), which allows the recording of high resolution STM images as well as of reliable cyclic voltammograms (even simultaneously) within the same electrochemical cell of 2.5 cm^3^ volume. This direct combination of STM and cyclic voltammetry (CV) permits a precise correlation of the obtained STM images to features in the corresponding cyclic voltamogram. A detailed description of this experimental setup is given in [59,67].

For the preparation of the organic films, a chloride precovered Cu(100) or Cu(111) single crystal was used as substrate, respectively. Initial CV and STM measurements were carried out several times in the supporting electrolyte (10 mM HCl) in order to obtain very well ordered and flat surfaces [18,59,67]. For the adsorption of the organic films on top of the chloride terminated electrode surfaces, the pure supporting electrolyte was substituted by a solution containing either the individual organic molecules, namely Dibenzylviologen (DBV) or 5,10,15,20-Tetrakis (4-trimethyl ammonium phenyl) porphyrin tetra p-toluenesulfonate) (H_2_TTMAPP) or a mixture of both species at a potential between −50 and +50 mV *vs*. RHE.

## 4. Summary

Electrochemical and structural features of layers of adsorbed viologen, porphyrin and a mixture of both these molecules have been addressed in this report by employing a combination of cyclic voltametry, and *in situ* scanning tunneling microscopy as well as referring to *ex situ* X-ray photoelectron spectroscopy data. While the viologen molecules undergo two single-electron reduction steps from DBV^2+^ to the corresponding radical mono-cationic DBV^•+^ and the uncharged DBV^0^ species, the [H_2_TTMAPP]^4+^ cations can accept up to six electrons in total within the potential window of the copper electrodes. On both electrode surfaces, chloride pre-covered Cu(100) and Cu(111) respectively, ordered layers of viologen and porphyrin are formed spontaneously. The observed and characterized structures reveal a significant influence of the molecular redox-state, and the symmetry of the substrates on the self-assembled layers, their inner conformation as well as phase transitions. As a result, detailed structure models are derived and are discussed in terms of the prevailing electrostatic interactions.

## References

[1] Lehn J.M. (1995). Supramolecular Chemistry: Concepts and Perspective.

[2] Lehn J.M. (1988). Supramolecular chemistry—Scope and perspectives molecules, supermolecules, and molecular devices. Angew. Chem. Int. Ed. Engl.

[3] Vögtle F (1991). Supramolecular Chemistry: An Introduction.

[4] Kaifer A.E., Gomez-Kaifer M (1999). Supramolecular Electrochemistry.

[5] Guillaud G., Simon J., Germain J.P. (1998). Metallophthalocyanines: Gas sensors, resistors, and field effect transistors. Coord. Chem. Rev..

[6] Collman J.P., Wagenknecht P.S., Hutchison J.E. (1994). Molecular catalysts for multielectron redox reactions of small molecules: The “Cofacial metallodiporphyrin” approach. Angew. Chem. Int. Ed. Engl.

[7] Balzani V (2001). Electron Transfer in Chemistry.

[8] Jasinski R. (1965). Cobalt phthalocyanine as a fuel cell cathode. J. Electrochem. Soc.

[9] Alt H., Binder H., Sandstede G. (1973). Mechanism of the electrocatalytic reduction of oxygen on metal chelates. J. Catal.

[10] Savy M., Andro P., Bernard C., Magner G. (1973). Etude de la reduction de l’oxygene sur les phtalocyanines monomeres et polymeres-I. principes fondamentaux, choix de l’ion central. Electrochim. Acta.

[11] Otsuki J., Nagamine E., Kondo T., Iwasaki K., Asakawa M., Miyake K. (2005). Article surface patterning with two-dimensional porphyrin supramolecular arrays. J. Am. Chem. Soc.

[12] Tao N.J., Cardenas G., Cunha F., Shi Z. (1995). *In situ* STM and AFM study of protoporphyrin and iron(III) and zinc(II) protoporphyrins adsorbed on graphite in aqueous solutions. Langmuir.

[13] Tao N.J. (1996). Probing potential-tuned resonant tunneling through redox molecules with scanning tunneling microscopy. Phys. Rev. Lett.

[14] He Y., Ye T., Borguet E. (2002). Porphyrin self-assembly at electrochemical interfaces: Role of potential modulated surface mobility. J. Am. Chem. Soc.

[15] He Y., Ye T., Borguet E. (2006). Adsorption and electrochemical activity: An *in situ* electrochemical scanning tunneling microscopy study of electrode reactions and potential-induced adsorption of porphyrins. J. Phys. Chem. B.

[16] Ogaki K., Batina N., Kunitake M., Itaya K. (1996). *In situ* scanning tunneling microscopy of ordering processes of adsorbed porphyrin on iodine-modified Ag(111). J. Phys. Chem. B.

[17] Sashikata K., Sutaga T., Sugimasa M., Itaya K. (1998). *In situ* scanning tunneling microscopy observation of a porphyrin adlayer on an iodine-modified Pt(100) electrode. Langmuir.

[18] Kunitake M., Batina N., Itaya K. (1995). Self-organized porphyrin array on iodine-modified Au(111) in electrolyte solutions: *In situ* scanning tunneling microscopy study. Langmuir.

[19] Wan L.J., Shundo S., Inukai J., Itaya K. (2000). Ordered adlayers of organic molecules on sulfur-modified Au(111): *In situ* scanning tunneling microscopy study. Langmuir.

[20] Nguyen T.M.H., Wandelt K., Broekmann P. (2008). Stable anion–cation layers on Cu(111) under reactive conditions. J. Phys. Chem. C.

[21] Hai N.T.M., Gašparović B., Wandelt K., Broekmann P. (2007). Phase transition in ordered porphyrin layers on iodide modified Cu(111): An EC-STM study. Surf. Sci.

[22] Xu B., Tao N.J. (2003). Measurement of single-molecule resistance by repeated formation of molecular junctions. Science.

[23] Haiss W., Zaling H.V., Higgins S.J., Bethell D., Horbenreich H., Schiffrin D.J., Nichols R.J. (2003). Redox state dependence of single molecule conductivity. J. Am. Chem. Soc.

[24] Haiss W., Nichols R.J., van Zaling H., Higgins S.J., Bethell D., Schiffrin D. (2004). Measurement of single molecule conductivity using the spontaneous formation of molecular wires. J. Phys. Chem. Chem. Phys.

[25] Li Z., Han B., Meszaros G., Pobelov I., Wandlowski Th., Blaszczyk A., Mayor M. (2006). Two-dimensional assembly and local redox-activity of molecular hybrid htructures in an electrochemical environment. Faraday Discuss..

[26] Imahori H., Norieda H., Yamada H., Nishimura Y., Yamazaki I., Sakata Y., Fukuzumi S. (2001). Light harvesting and photocurrent generation by gold electrodes modified with mixed self-assembled monolayers of boron-dipyrrin and ferrocene-porphyrin-fullerene triad. J. Am. Chem. Soc.

[27] Bird C.L., Kuhn A.T. (1981). Electrochemistry of the viologens. Chem. Soc. Rev.

[28] Monk P.M.S. (1998). The Viologens: Physicochemical Properties, Synthesis and Applications of the Salts of 4,4′-bipyridines.

[29] Dretschkow Th., Wandlowski Th. (1999). 2,2′-Bipyridine on Au(111)—A novel order/disorder adlayer transition. Electrochim. Acta.

[30] Diao Y.X., Han M.J., Wan L.J., Itaya K., Uchida T., Miyake H., Yamakata A., Osawa M. (2006). Adsorbed structures of 4,4′-bipyridine on Cu(111) in acid studied by STM and IR. Langmuir.

[31] Beckers E.H.A., Meskers S.C.J., Schenning A.P.H.J., Chen Z., Wurthner K., Janssen R.A.J. (2004). Charge separation and recombination in photoexcited oligo(p-phenylene vinylene): Perylene bisimide arrays close to the marcus inverted region. J. Phys. Chem. A.

[32] Hipps K.W., Scudiero L., Barlow D.E., Cooke M.P. (2002). A self-organized 2-dimensional bifunctional structure formed by supramolecular design. J. Am. Chem. Soc..

[33] Scudiero L., Hipps K.W., Barlow D.E. (2003). A Self-organized two-dimensional bimolecular structure. J. Phys. Chem. B.

[34] Yoshimoto S., Higa N., Itaya K. (2004). Two-dimensional supramolecular organization of copper octaethylporphyrin and cobalt phthalocyanine on Au(111): Molecular assembly control at an electrochemical interface. J. Am. Chem. Soc.

[35] Suto K., Yoshimoto S., Itaya K. (2003). Two-dimensional self-organization of phthalocyanine and porphyrin: Dependence on the crystallographic orientation of Au. J. Am. Chem. Soc.

[36] Kobayashi K., Fujisaki F., Yoshimina T., Nik K. (1986). An analysis of the voltammetric adsorption waves of methyl viologen. Bull. Chem. Soc. Jpn.

[37] Arihara K., Ohsaka T., Kitamura F. (2002). Characteristic cyclic voltammograms of alkyl viologens at single crystal gold electrodes. Phys. Chem. Chem. Phys.

[38] Pham D.T., Gentz K., Zörlein C., Nguyen T.M.H., Tsay S.L., Kirchner B., Kossmann S., Wandelt K., Broekmann P. (2006). Surface redox chemistry of adsorbed viologens on Cu(100). New J. Chem.

[39] Pham D.T. (2011). Self-Assembly of Viologen Molecules at Metal/Electrolyte Interfaces under Non-Reactive and Reactive Conditions. Ph.D. Dissertation.

[40] Suggs D.W., Bard A.J. (1995). Scanning tunneling microscopic study with atomic resolution of the dissolution of Cu(100) electrodes in aqueous chloride media. J. Phys. Chem. B.

[41] Vogt M.R., Lachenwitzer A., Magnussen O.M., Behm R.J. (1998). *In situ* STM study of the initial stages of corrosion of Cu(100) electrodes in sulfuric and hydrochloric acid solution. Surf. Sci.

[42] Magnussen O.M. (2002). Ordered anion adlayers on metal electrode surfaces. Chem. Rev.

[43] Ehlers C.B., Stickney J.L. (1989). Surface chemistry of electrodes: Cu(111) in aqueous HCl. J. Vac. Sci. Technol.

[44] Kruft M., Wohlmann B., Stuhlmann C., Wandelt K (1997). Chloride adsorption on Cu(111) electrodes in dilute HCl solutions. Surf. Sci..

[45] Wilms M., Broekmann P., Kruft M., Stuhlmann C., Wandelt K. (1998). STM investigation of step orientation and surface dynamics of Cu(111) in hydrochloric acid electrolyte. Appl. Phys.

[46] Peljhan S., Kokalj A. (2009). Adsorption of Chlorine on Cu(111): A density-functional theory study. J. Phys. Chem. C.

[47] Inukai J., Osawa Y., Itaya K. (1998). Adlayer structures of chlorine, bromine, and iodine on Cu(111) electrode in solution: *In situ* STM and *ex situ* LEED studies. J. Phys. Chem. B.

[48] Langhus D.L., Wilson G.S. (1979). Analysis of time dependent spectra generated from spectro electrochemical experiments. Anal. Chem.

[49] Heumann S., Hai N.T.M., Broekmann P., Wandelt K., Zajonz H., Dosch H., Renner F. (2006). X-ray diffraction and STM study of reactive surfaces under electrochemical control: Cl and I on Cu(100). J. Phys. Chem. B.

[50] Migani A., as F. (2006). A systematic study of the structure and bonding of halogens on low-index transition metal surfaces. J. Phys. Chem. B.

[51] Tolentino H.C.N., De Santis M., Gauthier Y., Langlais V. (2007). Chlorine chemisorption on Cu(001) by surface X-ray diffraction: Geometry and substrate relaxation. Surf. Sci.

[52] Batina N., Kunitake M., Itaya K. (1996). Highly ordered molecular arrays formed on iodine-modified Au(111) in solution: *in situ* STM imaging. J. Electroal. Chem.

[53] Broekmann P., Wilms M., Kruft M., Stuhlmann C., Wandelt K. (1999). *In situ* STM investigation of specific anion adsorption on Cu(111). J. Electroal. Chem.

[54] Pham D.T., Tsay S.L., Gentz K., Zorlein C., Kossmann S., Stay J.S., Kirchner B., Wandelt K., Broekmann P. (2007). Quasi reversible chloride adsorption/desorption through a polycationic organic film on Cu(100). Phys. Chem. C.

[55] Safarowsky C., Wandelt K., Broekmann P. (2004). Formation of supramolecular cavitands on copper electrode surfaces. Langmuir.

[56] Breuer S., Pham D.T., Huemann S., Gentz K., Zoerlein C., Hunger R., Wandelt K., Broekmann P. (2008). SXPS studies of porphyrin-adsorption at copper/electrolyte interfaces. New J. Phys.

[57] Liu X., Neoh K.G., Zhao L.P., Kang E.T. (2002). Surface functionalization of glass and polymeric substrates via graft copolymerization of viologen in an aqueous medium. Langmuir.

[58] Phan T.H., Wandelt K (2013). Adsorption features and structural transition of dibenzyl viologen adlayer at electrolyte/Cu(111) interface under non-reactive and reactive conditions.

[59] Phan T.H. (2012). *In situ* Characterization of Self-assembled Organic Layers at Anion Modified Metal/Electrolyte Interfaces. Ph.D. Dissertation.

[60] Weck M., Dunn A.R., Matsumoto K., Coates G.W., Lobkoysky E.B., Grubbs R.H. (1999). Influence of perfluoroarene–arene interactions on the phase behavior of liquid crystalline and polymeric materials. Angew. Chem. Int. Ed.

[61] Dretschkow Th., Wandlowski Th. (1999). Structural studies of 2,2′-bipyridine on Au(100). J. Electroanal. Chem..

[62] Phan T.H., Wandelt K. (2013). Self-assembly of metal free porphyrin layers at copper-electrolyte interfaces: Dependence on substrate symmetry. Surf. Sci.

[63] Langhus D.L., Wilson G.S. (1979). Spectroelectrochemistry and cyclic voltammetry of the ee mechanism in a porphyrin diacid reduction. Anal. Chem.

[64] Neri B.P., Wilson G.S. (1972). Electrochemical studies of mesotetra(4-*N*-methylpyridyl)porphine in acid solution. Anal. Chem.

[65] Phan T.H., Wandelt K Porphyrin adsorption on a Cu(111) electrode surface: Potential dependent *in situ* STM studies.

[66] Phan T.H., Wandelt K (2013). *In situ* scanning tunneling microscopy of potential dependent bimolecular component self-assembly consisting of viologen and porphyrin at an electrolyte/electrode interface.

[67] Hai N.T.M. (2011). Preparation and Characterization of Copper-Iodide Thin Films and Organic Supramolecular Layers at Copper/Electrolyte Interfaces. Ph.D. Dissertation.

